# Dietary intake of vitamin D in a northern Canadian Dené First Nation community

**DOI:** 10.3402/ijch.v72i0.20723

**Published:** 2013-08-05

**Authors:** Joyce Slater, Linda Larcombe, Chris Green, Caroline Slivinski, Matthew Singer, Lizette Denechezhe, Chris Whaley, Peter Nickerson, Pamela Orr

**Affiliations:** 1 Department of Human Nutritional Sciences, University of Manitoba, Winnipeg, MB, Canada; 2 Departments of Medical Microbiology, Medicine, and Community Health Sciences, University of Manitoba, Winnipeg, MB, Canada; 3 Department of Community Health Sciences, University of Manitoba, Winnipeg, MB, Canada; 4 General Delivery, Lac Brochet, MB, Canada; 5 Faculty of Medicine, University of Manitoba, Winnipeg, MB, Canada

**Keywords:** vitamin D, First Nations, indigenous, Aboriginal, diet, nutrition, food security

## Abstract

**Background:**

Increased awareness of the wide spectrum of activity of vitamin D has focused interest on its role in the health of Canada's Aboriginal peoples, who bear a high burden of both infectious and chronic disease. Cutaneous vitamin D synthesis is limited at northern latitudes, and the transition from nutrient-dense traditional to nutrient-poor market foods has left many Canadian Aboriginal populations food insecure and nutritionally vulnerable.

**Objective:**

The study was undertaken to determine the level of dietary vitamin D in a northern Canadian Aboriginal (Dené) community and to determine the primary food sources of vitamin D.

**Design:**

Cross-sectional study.

**Methods:**

Dietary vitamin D intakes of 46 adult Dené men and women were assessed using a food frequency questionnaire and compared across age, gender, season and body mass index. The adequacy of dietary vitamin D intake was assessed using the 2007 Adequate Intake (AI) and the 2011 Recommended Dietary Allowance (RDA) values for Dietary Reference Intake (DRI).

**Results:**

Mean daily vitamin D intake was 271.4 IU in winter and 298.3 IU in summer. Forty percent and 47.8% of participants met the vitamin D 1997 AI values in winter and summer, respectively; this dropped to 11.1 and 13.0% in winter and summer using 2011 RDA values. Supplements, milk, and local fish were positively associated with adequate vitamin D intake. Milk and local fish were the major dietary sources of vitamin D.

**Conclusions:**

Dietary intake of vitamin D in the study population was low. Only 2 food sources, fluid milk and fish, provided the majority of dietary vitamin D. Addressing low vitamin D intake in this population requires action aimed at food insecurity present in northern Aboriginal populations.

Food insecurity, defined as insufficient physical and/or economic access to adequate quantities of safe and nutritious food required to maintain health, is a significant public health concern for the Aboriginal (First Nations, Metis and Inuit) peoples of Canada (1–7). It is accompanied by overt as well as “hidden” hunger, the latter characterised by deficiencies in essential vitamins and micronutrients in those whose dietary intake is inexpensive and filling but not nutritious (8).

Vitamin D represents one of the many nutrients at risk in Canada's Aboriginal communities which are experiencing transition from a traditional lifestyle of hunting, fishing and gathering to a market-based economy (9–13). The wide spectrum of activity of vitamin D, not only on calcium and bone metabolism but also on the immune and cardiovascular systems, glucose metabolism and the regulation of cell proliferation (14), has stimulated a renewed interest in its role in the health and illness of indigenous people.

In the absence of national data, several recent regional studies have reported substandard serum levels of 25-hydroxycholecalciferol [25(OH)D] among Aboriginal Canadians, along with case reports of rickets and observations of elevated fracture risk associated with low bone mineral density among First Nations women (9, 10, 15, 16). Aboriginal Canadians experience an elevated incidence and prevalence of both infectious and chronic diseases, including tuberculosis and other respiratory infections, diabetes, collagen vascular diseases and selected malignancies (17). The potential role of vitamin D deficiency in the aetiology of these morbidities, and the opportunity, if any, for preventive and/or therapeutic intervention, is currently unknown.

Diminished solar ultraviolet B (UVB) radiation in northern latitudes results in limited cutaneous synthesis of vitamin D during northern winter months (from September to April) among Canadians who live in southern regions of the country (the most southern latitude being 42°), but especially among those Aboriginal peoples who live in more northern regions of the country (18). In terms of dietary sources, there are relatively few foods containing vitamin D (e.g. fish oils, eggs and fortified foods such as milk, margarine). However, prevalent food insecurity in many of Canada's Aboriginal communities, fuelled by high market food prices, high shipping costs in remote regions, low employment and poverty, has resulted in limited access to foods rich in vitamin D and other essential nutrients (2, 5, 12, 13, 19). Although several non-market traditional (or “country”) foods are good sources of vitamin D, consumption varies greatly among and within populations due to availability, cost of hunting and fishing, and concerns about environmental contamination (12, 19).

In this study, we describe the dietary vitamin D intake of residents in a Dené community in northern Canada, by season and with reference to age, gender, body mass index and food source. The Dené are First Nations peoples of the Athapaskan language group. The people of Lac Brochet (Denésuline First Nation), a small northern community in the province of Manitoba, represent a unique subset of Dené people who have lived in relative isolation and have preserved their cultural distinctiveness (20). They have struggled to maintain their traditional caribou hunting and fishing practices, despite economic, environmental, social and political challenges (20, 21).

Over the past 7 years, the community has engaged in community-based participatory research exploring the biologic and social determinants of health and illness among their people. The community has experienced high rates of infectious diseases, including epidemic tuberculosis (22). Determinants of health in Lac Brochet include inadequate housing and high rates of poverty and unemployment (21–23). In addressing these challenges, the community and its leadership have engaged in projects to identify and promote those aspects of their past and current way of life that serve to sustain health. It is hoped that the results may help the community and program/policymakers better understand and ameliorate the nutritional vulnerabilities of this population.

## Methods

### Recruitment of participants

In the winter and summer of 2010, dietary intake of vitamin D-containing foods was assessed in a population self-identifying as Dené in the community of Lac Brochet (Northlands Denésuline First Nation). This community, with a population of 604, is located at 58°N in Manitoba, Canada. The study was designed, implemented and analysed, and knowledge translation took place through a partnership between the community and the University of Manitoba. Ethical approval was granted by the University of Manitoba Ethics Research Board and the Band Chief and Council, and Aboriginal research principles of ownership, control, access and possession (OCAP) were followed (24).

In consultation with the community, and in accordance with their wish, recruitment of participants was performed through convenience rather than random sampling. Participants were eligible for inclusion if they were 18 years of age or older, gave informed consent, self-identified as Dené, able to participate for the duration of the study and committed to avoid taking vitamin D supplements >600 IU/day. Individuals taking a low dose of vitamin D (≤600 IU/day) were allowed to participate in the study, in order to more accurately reflect the health behavior of the general community population. Supplementary vitamin D intake was added to the calculation of dietary intake.

Exclusion criteria included the use of vitamin D supplements >600 IU/day for 3 months prior to study onset, clinical evidence of infection at the time of enrolment, first degree kinship with an individual already enrolled, and immunosuppressive medical condition or use of immunosuppressive medication, including systemic steroids.

A total of 105 community members were screened; 54 individuals met the study criteria. Forty-six participants completed the study: 2 individuals moved away from the community permanently, 2 were temporarily absent during testing periods, 3 withdrew for personal reasons and 1 developed a serious inter-current illness precluding further study participation.

### Dietary assessment of vitamin D

A food frequency questionnaire (FFQ) specific for vitamin D-containing foods was used to assess vitamin D intake over the previous 3 months. The vitamin D-specific FFQ developed and validated by Wu and colleagues was adapted to include local vitamin D-containing foods, including wild foods (25). The FFQ was administered by 4 trained authors (LL, CS, MS and CW) and was field-tested in the community. Vitamin D-containing foods available through the local grocery store, that were not on the original list, and wild foods that contained vitamin D, were added.

The FFQ was administered twice to reflect potential seasonal differences in diet (February: FFQ-Winter; and September: FFQ-Summer). Respondents were also queried about intake of vitamin D-containing supplements. Nutrient analysis was conducted using the Canadian Nutrient File (CNF) (26). For wild foods for which there were no vitamin D values in the CNF, the vitamin D value from a reasonable comparison food was used (beef for caribou tongue, heart, liver, kidney and fat).

### Data analysis

A database was created to capture frequency of consumption and portion size from the FFQs. Mean per person daily vitamin D intake by food item, as well as by vitamin supplementation, was calculated, and their contributions to total vitamin D intake were assessed using vitamin D values from the CNF (26).

Mean vitamin D intakes were calculated for each sub-group by winter and summer season and were compared using analysis of variance. Histogram plots and the Bartlett's Chi-square statistic were used to confirm that vitamin D intakes were normally distributed overall and had equal variances between comparison groups. The proportion of individuals achieving adequate vitamin D intakes was calculated according to whether they met the Institute of Medicine Dietary Reference Intakes (DRI) for both 1997 (also known as Adequate Intake, or AI) and 2011 (also known as Recommended Dietary Allowance, or RDA) (27). For the age categories 19–50, 51–70 and >70 years, the 1997 AI was 200, 400 and 600 IU/day, respectively, whereas the 2011 RDA was 600, 600 and 800 IU/day, respectively. Both the 1997 AI and 2011 RDA values were used in order to compare the changing standards, which are both in clinical use. Adequacy of vitamin D intake was compared between seasons, and within each season between male and female, age group (<40 and ≥40 years); and by measured body mass index (BMI) level, supplement use and fish/milk consumption. Relative risk ratios and associated confidence intervals were calculated for each within-FFQ sub-group.

## Results

Forty-six participants were recruited. One participant did not complete the first FFQ, but was included in the cohort. Mean age was 42 years (median 42, range 20–84 years); 25 were female and 21 were male. Mean BMI was 30.7 (median 28.6, range 21.1–47.0). Six participants were taking ≤400 IU vitamin D per day in a supplement during the winter and 8 in the summer.

Mean vitamin D intake in winter for the study cohort was 271.4 IU per day, compared with 298.3 IU per day (p=0.79) in summer (Table I). There was no significant difference between seasons. Participants taking low dose vitamin D supplements demonstrated significantly higher mean intakes in both winter (p=0.001) and summer (p=0.0039), compared to those who did not, when the supplementation was included in the calculation of their total intake. Consuming local fish 2 or more times per week resulted in significantly higher mean vitamin D intakes during the winter (p=0.0018) and summer (p=0.004) (Table I). Vitamin D intake was also significantly higher if a participant consumed 5 or more servings of milk per week in both winter (p=0.0006) and summer (p=0.0014). In all analysis of variance calculations, the Bartlett's Chi-square had a p-value greater than 0.05 which confirmed that comparison groups had similar variances. Histogram plots revealed that vitamin D intakes were left axis skewed, with the median value somewhat lower than the mean for both seasons. Since analysis of variance is considered to be reasonably robust to moderate departures from it assumptions (28), and because the generated p-values were either highly significant (less than 0.0018) or highly non-significant (greater than 0.05), it is unlikely that any false-positive or false-negative results were produced. Therefore, no data transformations or alternatives to the analysis of variance were deployed.

**Table I:** Analysis of dietary vitamin D intake (in International Units/day)

	FFQ-Winter			FFQ-Summer			
			
	n	Mean	Range	n	Mean	Range	p-Value^a^
All participants	45	271.4	33.2–855.5	46	298.3	27.0–970.3	0.7954
Vitamin D supplement							
Yes	6	534.2	310.3–731.5	8	494.4	225.9–724.0	0.5
No	39	231.0	33.27–855.5	38	256.5	27.0–970.3	0.8
p-Value^b^		0.0010			0.0039		
Fish							
≥2×/week	10	486.4	202.5–855.5	15	420.9	92.5–970.3	0.7
<2×/week	35	210.0	33.27–731.57	31	239.0	27.0–713.4	0.9
p-Value^b^		0.0018			0.0040		
Fluid milk							
≥5×/week	21	389.5	101.76–855.5	20	397.9	94.0–970.3	0.8
<5×/week	24	168.1	33.23–663.1	26	221.7	27.0–724.0	0.5
p-Value^b^		0.0006			0.0014		

a p-Values relate to mean comparisons between winter and summer.

b p-Values relate to comparison within a single season.

With reference to 1997 AI values, 40% of participants achieved adequate intakes in winter and 47.8% in summer (Table II). Thirty-six percent of females demonstrated adequate intakes in both winter and summer. More males had adequate intakes in summer (61.9%) than in winter (45%).

**Table II:** Proportion (%) and relative risk (RR) of participants meeting vitamin D 1997 Adequate Intake (AI) and 2011 Recommended Dietary Allowance (RDA)^a^, according to their winter and summer food frequency questionnaire (FFQ)

	FFQ-Winter						FFQ-Summer					
		
	1997 AI values			2011 RDA values			1997 AI values			2011 RDA values		
				
	%	RR	(95% CI)	%	RR	(95% CI)	%	RR	(95% CI)	%	RR	(95% CI)
All	40.0	n/a		11.1	n/a		47.8	n/a		13.0	n/a	
Sex												
Females	36.0	0.8000	0.39–1.63	8.0	0.5333	0.10–2.89	36.0	0.5815	0.31–1.08	16.0	1.6800	0.34–8.28
Male^c^	45.0			15.0			61.9			9.5		
Age group												
< 40 years	45.0	1.2500	0.61–2.55	10.0	0.8333	0.15–4.52	52.4	1.1905	0.65–2.17	9.5	0.5952	0.12–2.93
≥ 40 years^c^	36.0			12.0			44.0			16.0		
BMI^b^												
< 25	60.0	1.6500	0.84–3.25	10.0	0.8250	0.10–6.56	80.0	1.9429*	1.17–3.23	20.0	1.7000	0.36–7.96
≥ 25^c^	36.4			12.1			41.2			11.8		
Vitamin D supplement												
Yes	66.7	1.8571	0.92–3.76	16.7	1.6250	0.22–12.20	75.0	1.7813*	1.03–3.08	37.5	4.7500*	1.16–19.40
No^c^	35.9			10.3			42.1			7.9		
Fish												
≥ 2×/week	70.0	2.2273*	1.18–4.20	40.0	14.0000*	1.76–111.57	57.1	1.3061	0.72–2.38	28.6	4.5714	0.94–22.13
< 2×/week^c^	31.4			2.9			43.8			6.3		
Fluid milk												
≥ 5×/week	59.1	2.7182*	1.16–6.36	18.2	4.1818	0.51–34.56	75.0	2.7857*	1.41–5.51	25.0	6.5000	0.82–51.33
< 5×/week^c^	21.7			4.3			26.9			3.8		

a Reference 27.

b Body mass index.

c Reference for calculation of relative risk (RR).

* Significant at p<0.05.

A significantly greater proportion of individuals who took supplements or had normal BMI had adequate summer intakes. With reference to 2011 RDA values, only 11.1% of participants achieved adequate intakes in winter and 13% in summer.

**Fig. 1:**
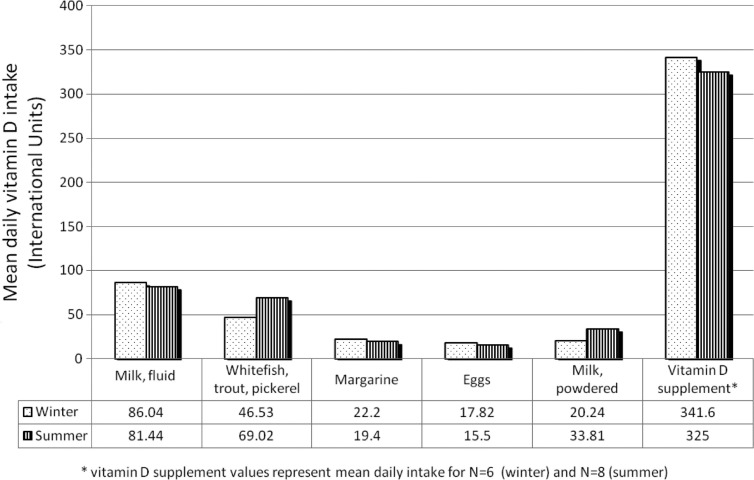
Daily mean intake of vitamin D, in winter and summer, according to source.

**Fig. 2:**
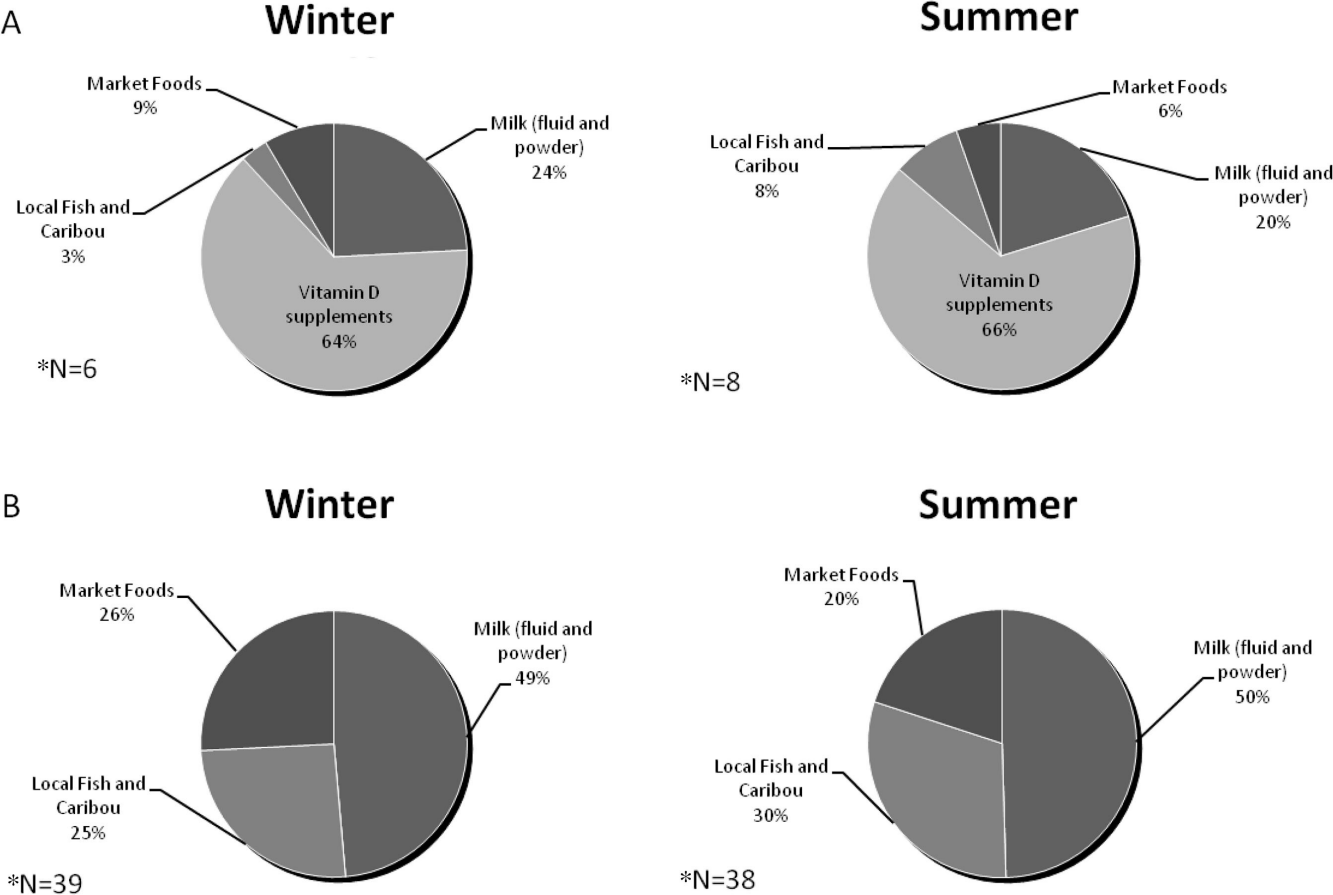
Seasonal percent of oral vitamin D intake by source for participants who took vitamin D supplements (A) and for those who did not (B). *FFQs obtained from 45 participants in winter and 46 in summer.

Five foods provided 69% of vitamin D for the study population, with the addition of supplements this increased to 87% (Fig. 1). Fluid milk was the most significant dietary source in the community, followed closely by local fish (flesh component). These 2 foods contributed 50% of dietary vitamin D. Margarine, eggs and powdered milk were secondary sources. Intake of foods did not vary by season with the exception of fish. Among participants who did not take vitamin D supplements, only 25% of total vitamin D intake came from country foods in the winter, and 31% in the summer (Fig. 2). Supplements contributed over 60% of the total vitamin D intake in the small number (n=6, winter; n=8, summer) of participants who took them.

Fewer than half of participants (n=20; 43%) drank 1 serving (250 ml) or more of milk 5 or more times per week. Less than one-third of participants consumed 1 serving (85 g) or more of fish 2 or more times per week.

## Discussion

Mean dietary intake of vitamin D in the study population was low; only 40% met the 1997 AI and only 13% met the 2011 RDA values. Only 2 food sources, fish and fluid milk, provided the majority of dietary vitamin D.

Mean vitamin D intake did not vary significantly by season, age or sex, indicating a relatively homogenous diet in the study population. Milk is available year-round in the community, as is local lake fish, an important traditional food source. A greater proportion of participants with normal BMI had adequate summer intakes, which may be indicative of an overall healthier diet in these individuals.

Fish and milk contribute significantly to dietary vitamin D intake in this northern population. However, less than one-third of the study group ate fish on a regular basis in either season. This could be due to the “nutrition transition” from country food sources to market foods, observed in many Canadian Aboriginal communities (3, 29, 30). Other explanations include participation in the global “nutrition transition” to high sugar/fat/salt processed foods, real or perceived contamination of wild foods with heavy metals and pollutants, and the high cost of equipment to fish and hunt (4, 30–32). These factors have led to profound food insecurity in many, northern communities (2, 4–7, 30–33).

Fluid milk was consumed regularly by less than half of study participants. Both economic and biologic factors are likely involved. The cost of milk in northern communities is very expensive. Previous studies have suggested that Aboriginal people have elevated rates of lactose intolerance, although the prevalence of this condition may not be as high as once thought (34–36). Forty-two percent of participants consumed milk regularly despite the cost: $12.99 for 4 litres at the time of the study, compared to $3.79 in Winnipeg, the southern capital of the province of Manitoba.

It is difficult to compare results found in this study with other Canadian studies of vitamin D status among different ethnic and age groups. Furthermore, not all studies accounted for seasonal variation. Nonetheless, the low dietary intakes in this study were consistent with those observed in other rural Aboriginal populations, and slightly higher than those reported for both off-reserve Aboriginals and non-Aboriginals in the Canadian Community Health Survey (9, 37, 38). This may be due to the consumption of traditional wild foods by the study cohort, in particular vitamin D-rich fish species. It is also important to note that low vitamin D dietary intake is prevalent throughout southern non-Aboriginal populations (15, 16).

The results of this study are consistent with other studies in similar populations. However, several study limitations are apparent. Dietary vitamin D is an imperfect marker of vitamin D status. However, in a companion study, the majority of this study cohort was found to have inadequate serum 25-OHD_3_ concentrations in winter (39). We also note that the sample size in this study, as in many studies in Canadian Aboriginal communities, is small. Reasons for this may include perceptions of research as exploitative and/or irrelevant to the struggles of daily life (40). The engagement of even a small cohort, in this context, is meaningful. Nevertheless, it is possible participants are not representative of all members of the community, and the generalisability of results to other northern Dené communities may also be limited.

Limitations also derive from the use of FFQs, which are imperfect instruments. The use of proxy (bovine) vitamin D content for caribou and fish components for which there are no published data is a source of potential error. It is possible that the local fish had different vitamin D levels that those found in the CNF. There were 2 fish species, jack and grayling, which were consumed in lesser quantities compared to other fish species in this community, and there were no vitamin D values or proxy values available. The community has expressed an interest in participating in further research to determine the vitamin D content of these and other traditional wild foods.

Policy measures to improve vitamin D intake require a Canada-wide population healthy strategy (11, 41–43). In northern and Aboriginal communities, cultural, economic and environmental challenges and opportunities are present. Communities should be supported in traditional harvesting and consumption of local fish when available (41, 43). Confidence in the safety of traditional food is required, as well as the means (knowledge and equipment) to ensure access for the current and future generations.

We recommend that the cost of fluid milk in northern communities should be subsidised to meet the cost in southern Canada (44). Fluid milk was first fortified with vitamin D in 1965 as a public health measure to prevent rickets, and remains the most common source of dietary vitamin D in Canada (38, 45). However, the cost of milk in northern and remote communities systematically excludes many northern Canadians from participating in this important public health strategy. This is further exacerbated by very high rates of poverty and ill-health; the median income in Lac Brochet in 2006 was $10,992, 60% of the adult population is unemployed, housing is crowded and in poor repair and tuberculosis is epidemic (21–23, 46). In 2010 the Government of Canada introduced the Nutrition North Program, which promotes and funds commercial and corporate food supplies (47). Resultant benefits are limited. The program subsidises fluid milk in northern Manitoba by 50 cents to $1 per kilogram (48); however this translates into a maximum of $4 per 4-litre container of milk, leaving the price still more than double the prices found in southern stores. In the absence of programs to support traditional non-commercial community-based hunting and fishing, access to traditional foods that contain vitamin D and other valuable nutrients remains tenuous (48).

Vitamin D supplementation may be an important adjunct to policy measures that address food security. However, addressing low vitamin D dietary intake in isolation will not solve the pervasive overt and hidden hunger that accompanies food insecurity in northern Aboriginal communities. Solutions must be holistic in a biologic and social sense, encompassing the broader issues of poverty, negative aspects of acculturation, environmental change and social justice, and mindful of Canada's commitment to the United Nations Universal Declaration of Human Rights (article 25) and Declaration on the Rights of Indigenous Peoples (articles 20–24, 29) (49, 50).
